# Dual-energy CT clot and peri-clot radiomics for predicting complete reperfusion and clinical outcome following endovascular therapy in acute ischemic stroke

**DOI:** 10.1186/s13244-026-02258-9

**Published:** 2026-03-30

**Authors:** Bi-Cong Yan, Li-Na Shi, Yi-Jia Xiong, Lin-Hui Zhao, Kai Sheng, Meng-Fei Wang, Zhuo Li, Bao-Hui Guan, Jing-Xuan Jiang, Yue-Hua Li

**Affiliations:** 1 Department of Diagnostic and Interventional Radiology, Shanghai Sixth People’s Hospital, Shanghai, China; 2 Faculty of Medical Imaging Technology, College of Health Science and Technology, Shanghai Jiao Tong University School of Medicine, Shanghai, China; 3 Department of Radiology, Renji Hospital, Affiliated with Shanghai Jiao Tong University, Shanghai, China; 4 School of Computer Science and Technology, Donghua University, Shanghai, China; 5 Shenyang University of Technology, Shenyang, China; 6 Department of Radiology, Affiliated Hospital of Nantong University, Nantong, China

**Keywords:** Stroke, Dual-energy CT, Endovascular therapy, Radiomics

## Abstract

**Objectives:**

Complete reperfusion is the optimal technical goal of endovascular therapy (EVT) and is closely linked to favorable outcomes in acute ischemic stroke (AIS). This study developed and validated clot- and peri-clot–based radiomics models on pre-interventional dual-energy CT angiography (DE-CTA) to predict complete reperfusion and clinical outcome after EVT.

**Materials and methods:**

A total of 371 patients from three centers were retrospectively enrolled and assigned to training (*n* = 154), test (*n* = 66), and validation (*n* = 151) cohorts. Radiomics features from clot and peri-clot regions were extracted, and three machine learning models—clot-based, peri-clot-based, and combined—were constructed. Model performance for predicting complete reperfusion and 90-day outcome was assessed using AUC.

**Results:**

Small, optimized feature subsets were selected for each model (11/11, 17/10, and 13/10 features for clot-based, peri-clot-based, and combined models for reperfusion and outcome prediction, respectively). For complete reperfusion, the peri-clot model showed the best performance with AUCs of 0.885 (95% CI: 0.834–0.937), 0.860 (95% CI: 0.771–0.948), and 0.847 (95% CI: 0.778–0.916) in the training, test, and validation cohorts, outperforming the clot-based (0.809, 0.759, 0.719) and combined models (0.867, 0.840, 0.820). A similar advantage was observed for outcome prediction, where the peri-clot model achieved the highest AUCs (0.854, 0.817, 0.850), exceeding the combined (0.839, 0.763, 0.804) and clot-based models (0.826, 0.709, 0.734).

**Conclusions:**

DE-CTA peri-clot radiomics provides superior prediction of both complete reperfusion and functional outcome after EVT, underscoring the clinical relevance of peri-clot microenvironment imaging and its potential to enhance pre-EVT patient selection and individualized prognostic evaluation.

**Trial registration:**

The trial registration number (Chinese Clinical Trial Registry, ChiCTR2400092800) and date of registration (2024.11.22) were retrospectively registered.

**Critical relevance statement:**

Peri-clot dual-energy computed tomography angiography radiomics outperformed clot-based models in multicenter external validation for predicting complete reperfusion and good 90-day functional outcome after endovascular therapy for acute ischemic stroke, supporting improved preprocedural risk stratification and patient selection.

**Key Points:**

- Complete reperfusion and functional recovery after endovascular thrombectomy remain hard to predict using thrombus features alone.
- Peri-clot radiomics on dual-energy computed tomography angiography achieved an external validation performance of 0.847 for complete reperfusion prediction.
- The same peri-clot model predicted 90-day functional outcome with 0.850 performance, supporting microenvironment-informed risk stratification.
- Peri-clot signatures consistently outperformed clot-only and combined models across multicenter cohorts, supporting robustness.

**Graphical Abstract:**

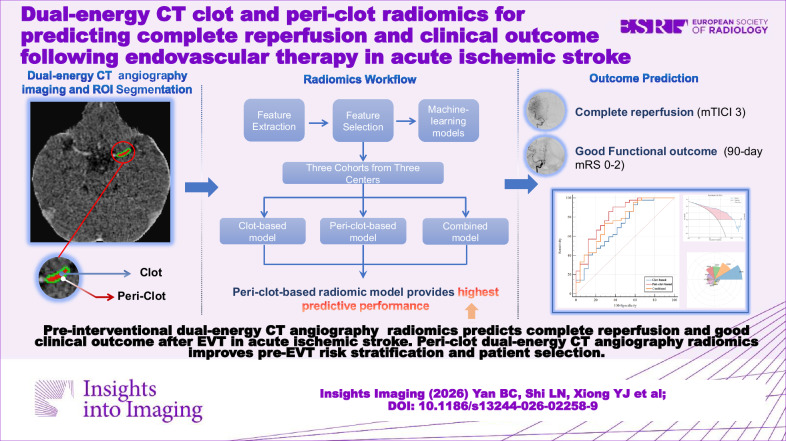

## Introduction

Stroke remains a leading cause of death and long-term disability worldwide. Endovascular thrombectomy (EVT) has revolutionized the management of acute ischemic stroke (AIS) due to large-vessel occlusion (LVO), offering substantially higher rates of successful revascularization compared with intravenous thrombolysis [1–3]. Achieving complete reperfusion—defined as a modified Thrombolysis in Cerebral Infarction (mTICI) grade of 3—has been firmly established as one of the strongest imaging predictors of favorable neurological recovery and reduced mortality [1, 4, 5]. Despite technical advances and increasing rates of mTICI 3 reperfusion, a considerable proportion of patients still experience poor functional outcomes, even after complete recanalization [6, 7].

Optimizing EVT outcomes requires a deeper understanding of the clot biology and the complex inflammatory milieu surrounding the occlusion. The mechanical response to thrombectomy is strongly influenced by clot composition and structure (e.g., burden/length and structural heterogeneity), which determine device–clot interaction, fragmentation risk, and the likelihood of distal embolization and microvascular obstruction [8]. In parallel, the peri-clot region represents a dynamic inflammatory and ischemic microenvironment. Experimental and clinical studies have shown that ischemic injury triggers a cascade of neuroinflammatory responses, endothelial dysfunction, and blood-brain barrier disruption, all of which promote microcirculatory failure and extension of irreversible tissue damage despite macrovascular recanalization [9–12]. Together, these observations suggest that both clot-intrinsic properties and peri-clot tissue status are critical, yet underappreciated, determinants of whether technically successful EVT ultimately translates into meaningful clinical recovery.

Dual-energy computed tomography (DECT) offers advantages over conventional CT by providing superior soft-tissue contrast and quantitative material decomposition [13]. By exploiting energy-dependent attenuation differences, DECT can distinguish iodine, calcium, and hemorrhage, refine clot density assessment, and detect residual or fragmented thrombus in AIS [14]. Radiomics is a rapidly evolving field for clinical problem-solving [15]. Clot-based radiomics studies have revealed and validated its effectiveness in predicting clot composition, the timing of stroke onset, and various other aspects of stroke [16–18]. Collectively, these properties make DECT an attractive imaging platform for simultaneously probing thrombus biology and the peri-clot parenchymal microenvironment, where early inflammatory and ischemic changes may critically shape microvascular reperfusion and subsequent clinical outcome.

This study develops and validates a dual-region radiomics framework leveraging pre-interventional DE-CTA to predict both complete reperfusion (mTICI 3) and clinical outcome following EVT in AIS. By jointly modeling DE-CTA-derived clot-intrinsic characteristics and peri-clot tissue signatures, we aim not only to deliver a clinically actionable tool for precision EVT triage but also to decode the pathophysiological determinants of reperfusion success beyond conventional angiographic metrics. This imaging-based strategy shifts the focus from vessel patency alone to a more comprehensive characterization of clot biology and perilesional vulnerability, enabling earlier and more accurate identification of patients most likely to achieve meaningful benefit from EVT and more realistic estimation of its technical and clinical success.

## Materials and methods

### Patient selection

The institutional review board of Shanghai Sixth People’s Hospital Affiliated to Shanghai Jiao Tong University School of Medicine approved this retrospective multicenter study (No. 2024-KY-203). A total of 386 consecutive adult patients (≥ 18 years old) with an anterior large-vessel occlusion (intracranial internal carotid artery (ICA), M1 segment of the middle cerebral artery, tandem occlusions) who were treated with EVT were retrospectively included from Feb. 2020 to Sept. 2024 at three centers: (A) Affiliated Hospital of Nantong University (Center A), (B) Shanghai Sixth People’s Hospital Affiliated to Shanghai Jiao Tong University School of Medicine (Center B), (C) Renji Hospital, School of Medicine, Shanghai Jiaotong University (Center C). The need for patient consent was waived in accordance with the ethical standards of the 1964 Declaration of Helsinki and its subsequent amendments.

Patients were included if they met the following criteria: (1) adults aged ≥ 18 years with AIS attributable to anterior circulation large-vessel occlusion who underwent EVT within 24 h of symptom onset; (2) underwent pre-interventional DE-CTA with diagnostic-quality imaging followed by immediate EVT; (3) documented onset-to-puncture time within 24 h; and (4) had complete demographic characteristics and validated clinical data with available follow-up data. Exclusion criteria comprised: (1) preprocedural intracranial hemorrhage, cerebral aneurysms, or arteriovenous malformations; (2) delayed intervention (onset-to-puncture > 24 h); (3) premorbid functional impairment (modified Rankin Scale score > 2); (4) suboptimal imaging quality due to motion/metal artifacts or technical limitations; and (5) incomplete medical records.

Demographic information (sex, age), occlusion location, medical history (including hypertension, diabetes, hyperlipidemia, atrial fibrillation, and coronary artery disease), and smoking status were obtained through retrospective review of existing clinical documentation.

**Fig. 1:**
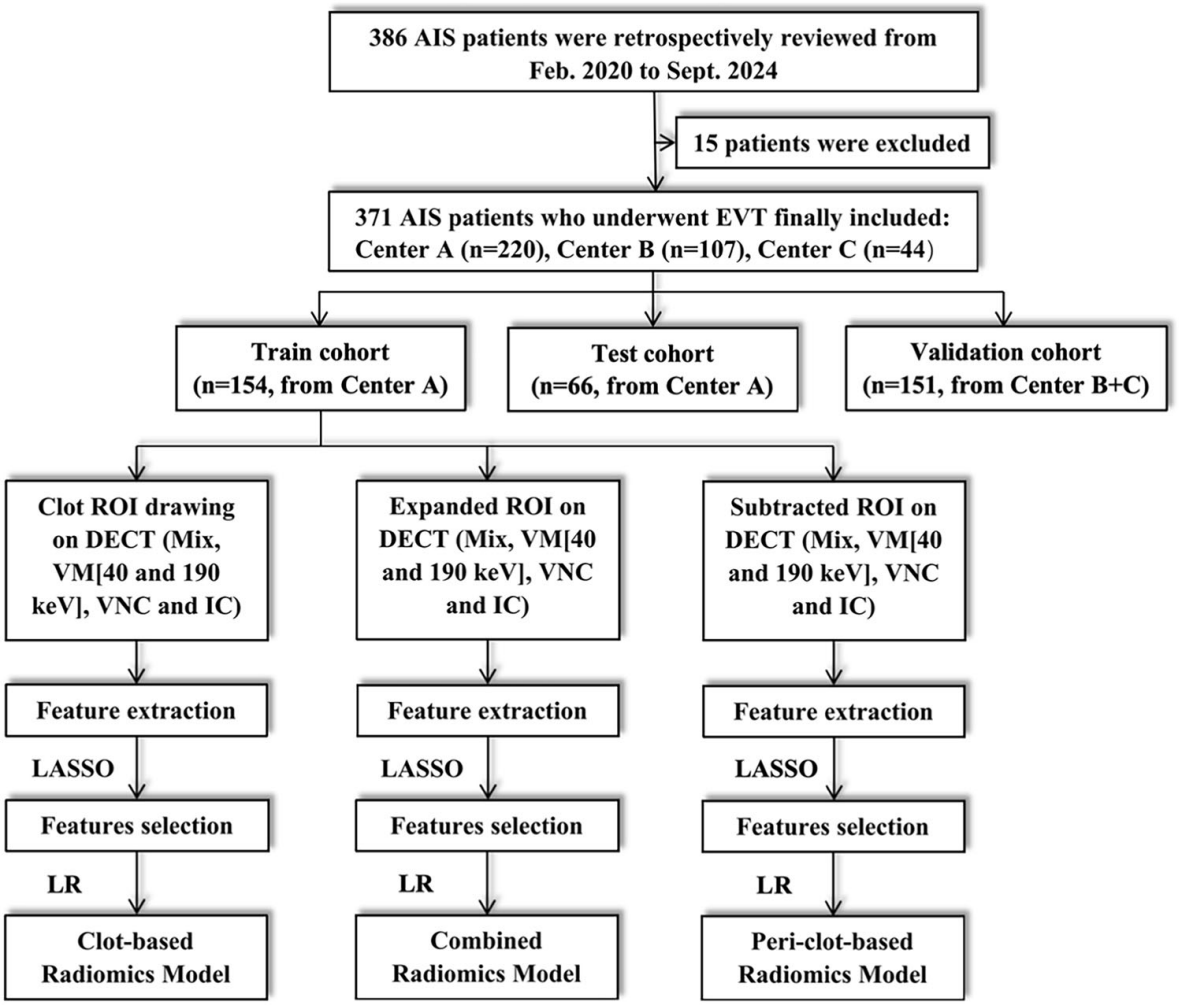
Flowchart showing the number (n) of patients included in the analysis and the models construction. LASSO, Least Absolute Shrinkage and Selection Operator; LR, logistic regression

The cohort was divided into train, test, and validation groups. Patients from Center A were randomly allocated (7:3 ratio) to train and test cohorts. Independent validation was performed using patients enrolled from Centers B and C combined. The complete enrollment pathway, including exclusion/inclusion filters, is presented in Fig. 1. Anonymized data and code scripts can be obtained for academic purposes by submitting a scientifically justified request to the corresponding author via institutional email.

### CT scanning and EVT performing

Admission imaging was performed using a dual-source DECT scanner (SOMATOM Force, Siemens Healthineers). Additional technical parameters and EVT procedures are provided in the supplementary materials. Dual-energy acquisition was applied for CTA (DE-CTA), whereas NCCT (and CTP, when performed) was acquired using routine single-energy protocols. For acute interpretation and EVT triage, mixed/blended CTA reconstructions were available immediately; material decomposition images were not awaited for decision-making.

Procedural success was evaluated through final angiographic assessment using the mTICI scale. Target vessel reperfusion was defined as achieving complete reperfusion (mTICI 3) flow restoration [19], as determined by final digital subtraction angiography (DSA) and verified by two independent senior interventionalists with > 10 years of subspecialty experience who were blinded to the clinical information and other index test results. Discrepancies were resolved by consensus with a third reviewer.

This study focused on two main outcomes: (1) procedural/angiographic outcome, defined as complete reperfusion (mTICI 3); and (2) clinical outcome, defined as the 90-day functional status assessed using the modified Rankin Scale (90-day mRS). The 90-day mRS was obtained through standardized follow-up by an independent stroke neurologist who was blinded to imaging findings and model outputs, with favorable outcome defined as mRS 0–2 and poor outcome as mRS 3–6.

### Imaging analysis and segmentation methodology

**Fig. 2:**
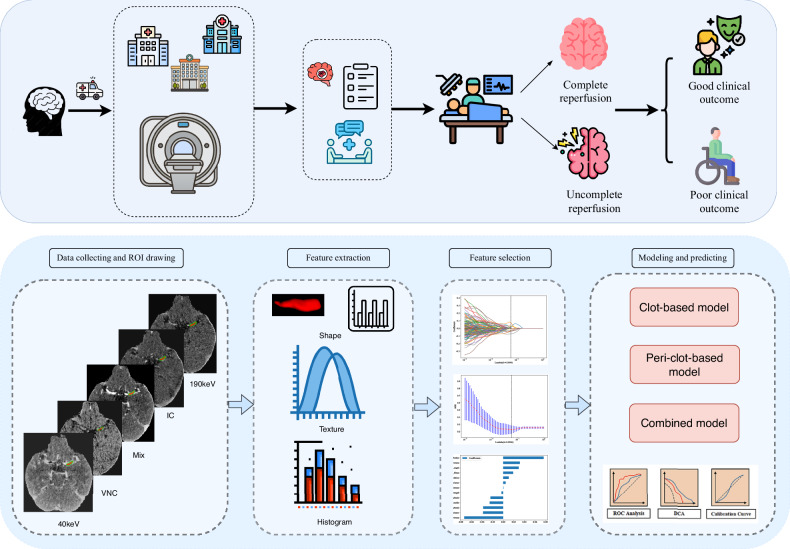
Workflow of the dual-energy CT angiography (DE-CTA)-based radiomics analysis. Patients with acute ischemic stroke undergo DE-CTA before endovascular therapy, after which reperfusion status and 90-day clinical outcome are assessed. Clot and peri-clot regions are segmented on DE-CTA-derived images, radiomics features (shape, texture, and intensity) are extracted, and relevant features are selected using LASSO. Three prediction models (clot-based, peri-clot-based, and combined) are then constructed and evaluated using ROC, decision curve, and calibration analyses

The radiomics workflow comprised four key phases: volumetric segmentation, feature extraction, feature selection, and predictive model development with validation (Fig. 2). Preprocessing included intensity clipping (0–600 Hounsfield Units) and spatial standardization achieved through isotropic resampling to 1 mm³ voxel dimensions.

Volumetric segmentation was performed using ITK-SNAP (v3.6.0; http://www.itksnap.org) with reference to dual-energy CT angiography (DE-CTA) images, as previously validated [17]. All DE-CTA-derived image sets from the same acquisition were used for analysis. Multi-image registration ensured spatial coherence across virtual monoenergetic (VM), virtual non-contrast (VNC), iodine concentration (IC), and mixed-energy (Mix) datasets within a standardized coordinate system.

Clot regions of interest (ROIs) were manually delineated on 0.5 mixing factor reconstructions (M_0.5: 50% low-kV/50% high-kV spectral fusion), guided by DE-CTA-derived image sets to exclude calcified components. A concentric peri-clot region was derived computationally through radial expansion (1 mm buffer zone) of the primary clot ROI using Python, with subsequent Boolean subtraction defining the peri-clot compartment, and termed peri-clot ROIs.

Interobserver variability was quantified through independent segmentation by two blinded neuroradiologists (> 5 years’ neuroimaging experience) in a 40-patient subset. To mitigate recall bias, repeat analyses were conducted with a 1-month washout interval, ensuring complete blinding to clinical outcomes during segmentation.

### Radiomics feature extraction and normalization

Following volumetric segmentation, radiomic features were extracted using the open-source PyRadiomics toolkit (version 3.0.1, https://pyradiomics.readthedocs.io/), including first-order statistics quantifying intensity distribution, shape-based descriptors characterizing 3D morphology, and texture features analyzing gray-level patterns. All features underwent z-score normalization (mean-centered with unit variance scaling) within the train cohort, with the resulting normalization parameters subsequently applied to both test and validation datasets to maintain consistent feature scaling.

### Feature selection and model construction

Feature selection was performed solely within the train cohort to ensure methodological rigor and avoid information leakage. To address feature redundancy, we implemented a two-step selection process for each prediction task: First, we applied the Mann–Whitney U test to screen all radiomics features, retaining only those demonstrating statistical significance (*p*-value < 0.05). Subsequently, we evaluated inter-feature relationships using Spearman’s rank correlation coefficients and iteratively removed the feature with the largest mean absolute correlation across the retained features in any pair with a correlation coefficient > 0.9.

For both study endpoints, which were complete reperfusion (mTICI 3) and the good clinical outcome (90-day mRS 0–2), we applied the least absolute shrinkage and selection operator (LASSO) regression within the train cohort to further reduce dimensionality and identify feature subsets specific to each outcome. Following LASSO selection, we developed the final radiomics signature for each endpoint using logistic regression (LR) with 5-fold cross-validation (see Supplementary Fig. S1 for the overall modeling workflow). The selected features were subsequently integrated to construct three distinct predictive models for each outcome: (1) a clot-based model, (2) a peri-clot-based model, and (3) a combined model, all developed using the LR methodology.

### Model validation, discrimination, and calibration

The predictive performance of the clot-based, peri-clot-based, and combined models was systematically evaluated in both test and validation cohorts. We additionally evaluated model performance separately in the tandem-lesion and non-tandem-lesion subgroups to assess their stability across different patient subpopulations. Model discrimination was assessed using receiver operating characteristic (ROC) analysis, with the area under the curve (AUC) serving as the primary metric of predictive accuracy. Model calibration was examined through calibration curves comparing predicted probabilities with observed outcomes in the train, test, and validation cohorts.

Comparative statistical analysis of model performance was performed using the DeLong test for paired ROC curves. Clinical utility was evaluated through decision curve analysis (DCA), which quantified the net benefit across a range of clinically relevant threshold probabilities. The study adheres to the Checklist for Evaluation of Radiomics Research (CLEAR) as a comprehensive reporting guideline for radiomics studies [20]. All validation procedures described above were performed separately for complete reperfusion (mTICI 3) and for good clinical outcome (90-day mRS 0–2), with clot-based, peri-clot-based, and combined models evaluated in parallel across all cohorts.

### Statistical analysis

All analyses were performed using IBM SPSS Statistics (version 21.0; IBM Corp.), MedCalc Statistical Software (version 18.2.1; MedCalc Software Ltd.), and FeAture Explorer Pro (FAE, version 0.3.5; https://github.com/salan668/FAE). Continuous variables were compared using Student’s *t*-test for normally distributed data and the Mann–Whitney U test for non-normally distributed data. Categorical variables were analyzed using either the chi-square test or Fisher’s exact test, as appropriate. Feature correlations were evaluated using Spearman’s rank correlation coefficients. Diagnostic performance was assessed through ROC curve analysis, with calculation of the AUC, sensitivity, specificity, positive predictive value (PPV), and negative predictive value (NPV) for predicting complete reperfusion following EVT. Comparative analysis of ROC curves was performed using the DeLong test. Clinical utility was evaluated through decision curve analysis (DCA). A two-tailed *p* < 0.05 was considered statistically significant for all analyses.

## Results

### Patient characteristics

A total of 386 patients were reviewed, and 15 (3.9%) patients were excluded due to inadequate image quality (*n* = 4), missing clinical data or clinical outcome (*n* = 7), pre-existing intracranial hemorrhage (*n* = 3), or treatment delay exceeding 24 h from symptom onset (*n* = 1). The final study cohort comprised 371 patients (220 [59.3%] from Center A, 107 [28.8%] from Center B, and 44 [11.9%] from Center C). Cohort allocation was performed with Center A patients randomly divided in a 7:3 ratio into the train (*n* = 154) and test cohorts (*n* = 66), while the independent validation cohort included 151 patients from Centers B and C.

Baseline demographic and clinical characteristics demonstrated no significant differences between patients achieving complete reperfusion versus non-complete reperfusion, or between those with favorable versus unfavorable 90-day clinical outcomes (all *p* > 0.05; Table 1). Detailed baseline characteristics, including vascular risk factors, procedural details and imaging parameters, are systematically presented in Table 1, with stratification by both reperfusion status and 90-day mRS outcome.

**Table 1:** Characteristics of included acute ischemic stroke patients

Characteristics	Train cohort (*n* = 154)				Test cohort (*n* = 66)				Validation cohort (*n* = 151)			
	All	Complete reperfusion	Non-complete reperfusion	*p*-value	All	Complete reperfusion	Non-complete reperfusion	*p*-value	All	Complete reperfusion	Non-complete reperfusion	*p*-value
Age (years, mean ± SD)	68.38 ± 11.74	69.11 ± 13.22	67.52 ± 9.75	0.084	69.00 ± 12.25	69.20 ± 12.27	68.68 ± 12.47	0.761	64.72 ± 13.50	63.46 ± 13.03	66.57 ± 14.08	0.165
NIHSS	10.34 ± 5.48	10.45 ± 5.01	10.21 ± 6.02	0.603	11.95 ± 5.84	13.10 ± 6.45	10.08 ± 4.16	0.073	12.58 ± 6.99	12.92 ± 7.09	12.08 ± 6.86	0.365
Gender, *n* (%)				1.0				0.902				0.501
Female	56 (36.36)	30 (36.14)	26 (36.62)		31 (46.97)	20 (48.78)	11 (44.00)		48 (31.79)	31 (34.44)	17 (27.87)	
Male	98 (63.64)	53 (63.86)	45 (63.38)		35 (53.03)	21 (51.22)	14 (56.00)		103 (68.21)	59 (65.56)	44 (72.13)	
Medical history, *n* (%)												
Atrial fibrillation	54 (35.06)	29 (34.94)	25 (35.21)	1.0	25 (37.88)	16 (39.02)	9 (36.00)	1.0	43 (28.48)	27 (30.00)	16 (26.23)	0.749
Smoke	35 (22.73)	19 (22.89)	16 (22.54)	1.0	9 (13.64)	6 (14.63)	3 (12.00)	1.0	44 (29.14)	24 (26.67)	20 (32.79)	0.529
Hypertension	91 (59.09)	46 (55.42)	45 (63.38)	0.403	45 (68.18)	28 (68.29)	17 (68.00)	1.0	83 (54.97)	48 (53.33)	35 (57.38)	0.746
Hyperlipidemia	7 (4.55)	1 (1.20)	6 (8.45)	0.078	3 (4.55)	1 (2.44)	2 (8.00)	0.658	6 (3.97)	4 (4.44)	2 (3.28)	1.0
Diabetes	35 (22.73)	16 (19.28)	19 (26.76)	0.362	22 (33.33)	13 (31.71)	9 (36.00)	0.929	44 (29.14)	23 (25.56)	21 (34.43)	0.320
Coronary heart disease	70 (45.45)	34 (40.96)	36 (50.70)	0.295	34 (51.52)	23 (56.10)	11 (44.00)	0.484	49 (32.45)	28 (31.11)	21 (34.43)	0.803
Clot location				0.233				0.330				0.476
MCA	89 (57.79)	53 (63.86)	36 (50.70)		49 (74.24)	33 (80.49)	16 (64.00)		95 (62.91)	60 (66.67)	35 (57.38)	
ICA	43 (27.92)	19 (22.89)	24 (33.80)		15 (22.73)	7 (17.07)	8 (32.00)		38 (25.17)	21 (23.33)	17 (27.87)	
MCA + ICA	22 (14.29)	11 (13.25)	11 (15.49)		2 (3.03)	1 (2.44)	1 (4.00)		18 (11.92)	9 (10.00)	9 (14.75)	
Clinical outcome				< 0.001				0.009				< 0.001
mRS 0–2	101 (65.6%)	71 (70.3%)	30 (29.7%)		42 (63.6%)	29 (69.0%)	13 (31.0%)		82 (70.4%)	57 (69.5%)	25 (30.5%)	
mRS 3–6	53 (34.4%)	19 (35.8%)	34 (64.2%)		24 (36.4%)	8 (33.3%)	16 (66.7%)		69 (29.6%)	24 (34.8%)	45 (65.2%)	

*ICA* internal carotid artery, *MCA* middle cerebral artery, *mRS* modified Rankin Scale, *NIHSS* National Institutes of Health Stroke Scale

### Data processing

**Fig. 3:**
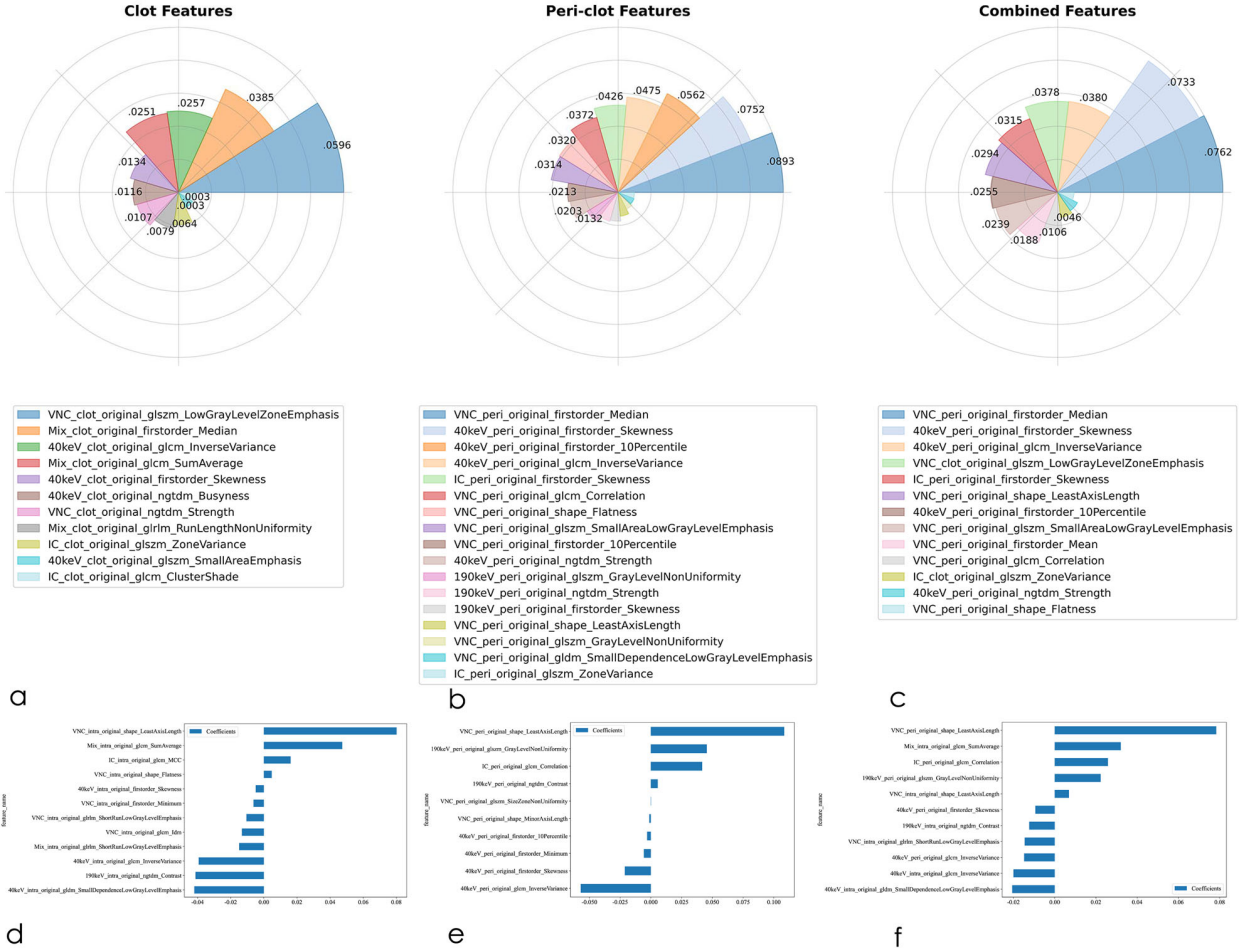
Radiomics signatures selected for the clot-based, peri-clot-based, and combined models using LASSO regression. The top row displays radar charts illustrating the relative contributions of selected radiomics features to each model’s signature. **a**–**c** The non-zero LASSO coefficients of features used to predict complete reperfusion (mTICI 3) for the clot-based (**a**), peri-clot-based (**b**), and combined (**c**) models. **d**–**f** The non-zero coefficients of features selected for predicting good clinical outcome (90-day mRS 0–2) for the clot-based (**d**), peri-clot-based (**e**), and combined (**f**) models. Across both prediction tasks, peri-clot-based models exhibit more diverse and higher-magnitude feature contributions, consistent with their superior performance across all cohorts

A comprehensive radiomics analysis was performed, with 107 distinct features extracted from the DE-CTA-derived image sets, yielding an initial pool of 535 quantitative features. Following rigorous feature selection, we identified 11 and 11 optimal radiomics features for predicting complete reperfusion and good clinical outcome in the clot-based model, 17 and 10 in the peri-clot-based model, and 13 and 11 in the combined model, respectively; the selected features and their coefficients are provided in Fig. 3. Feature selection and weighting were performed using LASSO logistic regression, with the resulting radiomics score (radscore) calculation methodology illustrated in the Supplementary Fig. S2 (detailed formula provided in Supplementary Material). Quality control assessments demonstrated excellent feature reproducibility, with both inter-rater and intra-rater intraclass correlation coefficients exceeding 0.75 across 40 independently analyzed regions of interest.

### Diagnostic performance and model comparison

**Fig. 4:**
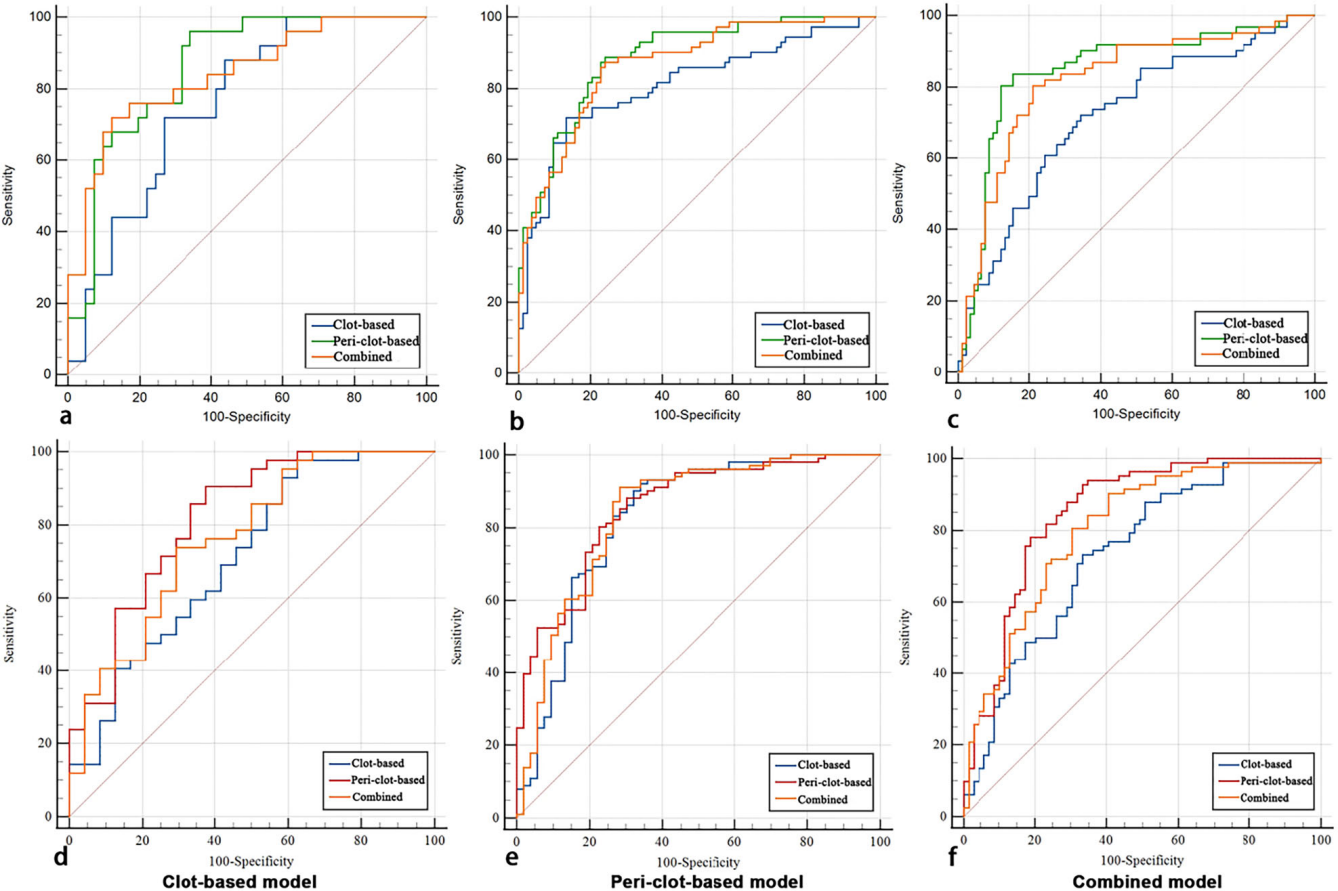
Receiver operating characteristic (ROC) curves of the clot-based, peri-clot-based, and combined radiomics models. **a**–**c** ROC curves for predicting complete reperfusion (mTICI 3) in the train (**a**), test (**b**), and validation (**c**) cohorts, respectively. **d**–**f** ROC curves for predicting good clinical outcome (90-day mRS 0–2) in the corresponding training (**d**), test (**e**), and validation (**f**) cohorts. In each panel, the performance of the clot-based, peri-clot-based, and combined models is displayed for direct comparison

**Fig. 5:**
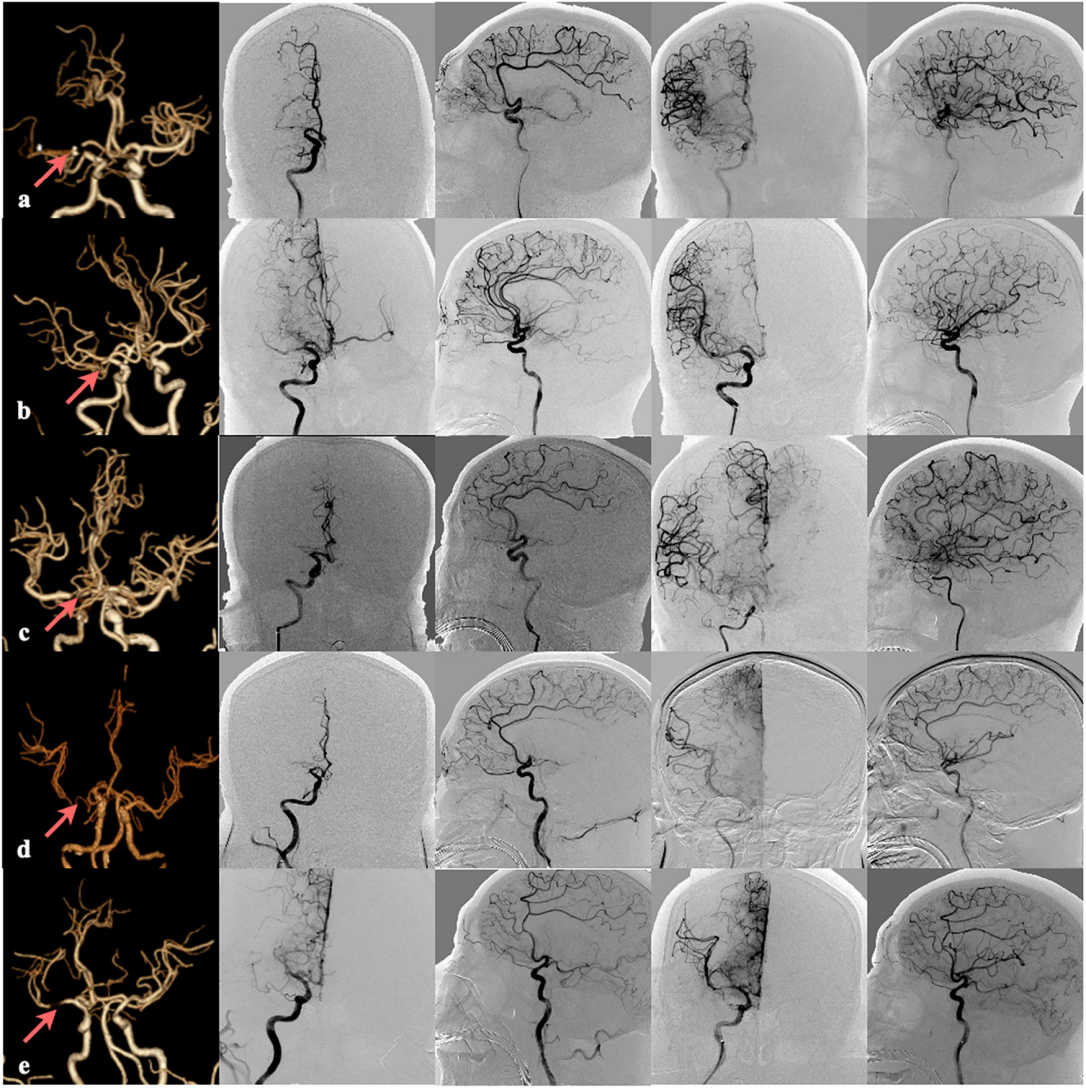
Representative cases of acute ischemic stroke with right-sided large-vessel occlusion treated by EVT in peri-clot model: **a** Proximal M1 occlusion with predicted and confirmed complete reperfusion with good clinical outcome (90-day mRS 0); **b** M1 occlusion with predicted and confirmed complete reperfusion with good clinical outcome (90-day mRS 1); **c** Terminal internal carotid artery occlusion with predicted non-complete but ultimately confirmed complete reperfusion with good clinical outcome (90-day mRS 1); **d** M1 segment terminus occlusion with predicted complete but ultimately non-complete reperfusion with poor clinical outcome (90-day mRS 4); and (**e**) M1-M2 junction occlusion with predicted and confirmed non-complete reperfusion with poor clinical outcome (90-day mRS 3). The first column shows CTA images with red arrows indicating the occlusion site; the second and third columns display pre-thrombectomy DSA (anteroposterior and lateral views); and the fourth and fifth columns demonstrate post-thrombectomy DSA (same projections) with corresponding reperfusion outcomes. EVT, endovascular therapy; CTA, computed tomography angiography; DSA, digital subtraction angiography

The peri-clot-based radiomics model (shown in Table 2) demonstrated superior discriminative ability across all cohorts, with AUC values of 0.885 (95% CI: 0.834–0.937) in the train cohort, 0.860 (95% CI: 0.771–0.948) in the test cohort, and 0.847 (95% CI: 0.778–0.916) in the validation cohort, significantly outperforming both the clot-based and combined models (all pairwise comparisons *p* < 0.05; Fig. 3). Notably, the combined model showed intermediate performance that was statistically superior to the clot-based model in all cohorts (all *p* < 0.05). Comprehensive diagnostic parameters, including sensitivity, specificity, PPV, and NPV, are detailed in Table 2. Optimal prediction thresholds derived from receiver operating characteristic analysis are presented in Fig. 4, while representative case examples illustrating model applications are shown in Fig. 5. In subgroup analyses of tandem-lesion and non-tandem-lesion patients, the peri-clot-based model consistently showed the highest AUC within each subgroup, supporting stable discrimination across patient subpopulations (Supplementary Tables S1, S2).

**Table 2:** Performance of the clot-, peri-clot-based and combined models predicting complete reperfusion across cohorts

Cohorts	Models	SEN	SPE	ACC	PPV	NPV	Precision	Recall	F1	Threshold	AUC (95% CI)	*p*-value
Train cohort	Clot-based model	0.704	0.867	0.792	0.820	0.774	0.820	0.704	0.758	0.505	0.809 (0.738–0.881)	0.017^#^
	Peri-clot-based model	0.873	0.759	0.812	0.756	0.875	0.756	0.873	0.810	0.368	0.885 (0.834–0.937)	0.910*
	Combined model	0.859	0.759	0.805	0.753	0.863	0.753	0.859	0.803	0.385	0.867 (0.811–0.923)	0.003^^^
Test cohort	Clot-based model	0.680	0.732	0.712	0.607	0.789	0.607	0.680	0.642	0.455	0.759 (0.644–0.874)	< 0.001^#^
	Peri-clot-based model	0.920	0.659	0.758	0.622	0.931	0.622	0.920	0.742	0.342	0.860 (0.771–0.948)	0.384*
	Combined model	0.680	0.878	0.803	0.773	0.818	0.773	0.680	0.723	0.565	0.840 (0.739–0.941)	< 0.001^^^
Validation cohort	Clot-based model	0.705	0.656	0.675	0.581	0.766	0.581	0.705	0.637	0.405	0.719 (0.636–0.803)	0.053^#^
	Peri-clot-based model	0.787	0.878	0.841	0.814	0.859	0.814	0.787	0.800	0.543	0.847 (0.778–0.916)	0.881*
	Combined model	0.787	0.789	0.788	0.716	0.845	0.716	0.787	0.750	0.489	0.820 (0.748–0.891)	0.020^^^

*SPE* specificity, *SEN* sensitivity, *ACC* accuracy, *PPV* positive predictive value, *NPV* negative predictive value, *AUC* area under the receiver operating characteristic curve, *CI* confidence interval

# The clot-based model’s AUC value compared with peri-clot-based model’s by Delong test

* The peri-clot-based model’s AUC value compared with combined model’s by Delong test

^ The combined model’s AUC value compared with clot-based model’s by Delong test

For the prediction of 90-day mRS, a similar pattern was observed (Table 3). The peri-clot-based model again provided the best discrimination, with AUCs of 0.854, 0.817, and 0.850 in the train, test, and validation cohorts, respectively. The combined model achieved intermediate AUCs (0.839, 0.763, and 0.804), whereas the clot-based model yielded the lowest values (0.826, 0.709, and 0.734) (shown in Fig. 4). DeLong tests indicated no significant differences among models in the train cohort (all *p* > 0.05), but peri-clot-based and combined models significantly outperformed the clot-based model in the test and validation cohorts (*p*-values ranging from 0.002 to 0.035). Full diagnostic metrics for clinical outcome prediction are presented in Table 3.

**Table 3:** Performance of the clot-, peri-clot-based and combined models predicting clinical outcome across cohorts

Cohorts	Models	SEN	SPE	ACC	PPV	NPV	Precision	Recall	F1	Threshold	AUC (95% CI)	*p*-value
Train cohort	Clot-based model	0.921	0.660	0.831	0.838	0.814	0.838	0.921	0.877	0.543	0.826 (0.749–0.904)	0.491^#^
	Peri-clot-based model	0.881	0.698	0.818	0.848	0.755	0.848	0.881	0.864	0.532	0.854 (0.792–0.916)	0.653*
	Combined model	0.911	0.717	0.844	0.860	0.809	0.860	0.911	0.885	0.563	0.839 (0.766–0.912)	0.500^^^
Test cohort	Clot-based model	0.976	0.375	0.758	0.732	0.900	0.732	0.976	0.837	0.334	0.709 (0.575–0.844)	0.102^#^
	Peri-clot-based model	0.905	0.625	0.803	0.809	0.789	0.809	0.905	0.854	0.548	0.817 (0.706–0.929)	0.316*
	Combined model	0.738	0.708	0.727	0.816	0.607	0.816	0.738	0.775	0.626	0.763 (0.641–0.885)	0.035^^^
Validation cohort	Clot-based model	0.732	0.667	0.702	0.723	0.676	0.723	0.732	0.727	0.697	0.734 (0.653–0.815)	0.002^#^
	Peri-clot-based model	0.927	0.667	0.808	0.768	0.885	0.768	0.927	0.840	0.520	0.850 (0.786–0.914)	0.095*
	Combined model	0.805	0.696	0.756	0.759	0.750	0.759	0.805	0.781	0.668	0.804 (0.733–0.875)	0.003^^^

*SPE* specificity, *SEN* sensitivity, *ACC* accuracy, *PPV* positive predictive value, *NPV* negative predictive value, *AUC* area under the receiver operating characteristic curve, *CI* confidence interval

# The clot-based model’s AUC value compared with peri-clot-based model’s by Delong test

* The peri-clot-based model’s AUC value compared with combined model’s by Delong test

^ The combined model’s AUC value compared with clot-based model’s by Delong test

### Model calibration and clinical utility

**Fig. 6:**
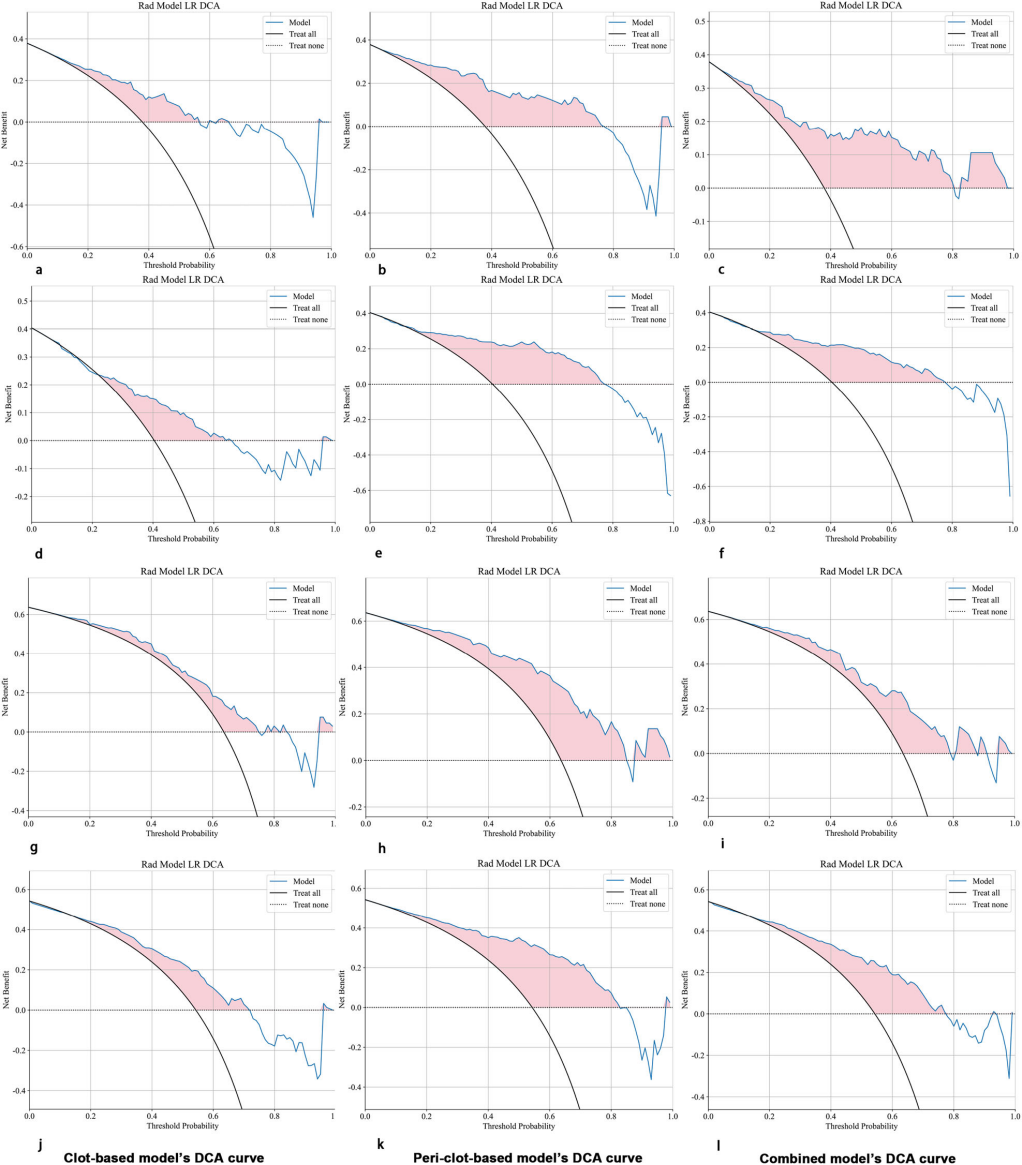
Decision curve analysis (DCA) of the clot-based, peri-clot-based, and combined radiomics models. The first and second rows (**a**–**c** and **d**–**f**) show DCA curves for predicting complete reperfusion (mTICI 3) in the test and validation cohorts, respectively, whereas the third and fourth rows (**g**–**i** and **j**–**l**) show the corresponding curves for predicting good clinical outcome (90-day mRS 0–2). Within each row, the left, middle, and right plots represent the clot-based, peri-clot-based, and combined models, respectively. Across prediction tasks and cohorts, all three radiomics models provide greater net benefit than the treat-all or treat-none strategies over a wide range of threshold probabilities, indicating their potential clinical utility in EVT decision-making

Calibration analysis demonstrated excellent agreement between predicted probabilities and observed outcomes for all models across the train, test, and validation cohorts (Supplementary Fig. S3), with no significant deviations from the ideal calibration line (Hosmer–Lemeshow test *p* > 0.05 for all models). Decision curve analysis (Fig. 6) revealed that both the peri-clot-based and combined models provided greater net benefit across a clinically relevant range of threshold probabilities compared to the clot-based model alone.

## Discussion

This multicenter study demonstrates that DE-CTA-derived peri-clot radiomics features provide superior predictive value for both complete reperfusion and clinical outcome in patients with acute ischemic stroke undergoing EVT. Across all cohorts, the peri-clot model consistently outperformed both clot-based and combined models, indicating that tissue characteristics surrounding the clot carry important prognostic information beyond the clot itself. These findings highlight the potential of peri-clot radiomic profiling as a valuable pre-interventional biomarker for optimizing EVT decision-making and anticipating patient prognosis.

Machine learning models incorporating clinical variables have demonstrated significant potential in predicting outcomes following EVT for AIS [21], thereby refining patient selection and treatment strategies. For instance, a study leveraging non-contrast CT clot composition analysis achieved robust performance in predicting EVT success (AUCs of 0.860 internally and 0.849 in an external validation cohort) [22]. A clot-based radiomics model with 9 features predicted successful first-attempt recanalization with thrombus aspiration (AUC 0.88) [23]. An end-to-end CTA-based deep learning pipeline achieved a test AUC of 0.70 using CTA alone, improving to 0.79–0.86 after adding treatment and clinical variables in outcome prediction after thrombectomy [24]. A structured comparison of representative machine learning/deep learning studies (inputs, endpoints, and AUCs) is provided in Supplementary Table S3. In this study, our optimized peri-clot-based model exhibited strong predictive performance for complete reperfusion (AUC 0.885 [95% CI: 0.834–0.937] in the train cohort) and also demonstrated favorable discriminative performance for clinical outcome, suggesting that peri-clot radiomics may capture tissue vulnerability relevant to both technical success and long-term recovery. This may be facilitated by DE-CTA-derived VNC and optimized VM reconstructions that enhance vascular attenuation and peri-clot tissue delineation [13, 25].

These findings collectively suggest that DE-CTA-derived peri-clot radiomics capture biologically meaningful information that links procedural and clinical outcomes. Complete reperfusion does not always translate into good functional recovery, likely reflecting downstream microvascular injury and inflammation despite angiographic success [26]. Mechanistically, inflammatory-permeability pathways have been associated with reperfusion quality after EVT, including circulating mediators such as IL-6 (reported to relate to futile reperfusion) [27], and neutrophil-to-lymphocyte ratio [28], MMP-9 (implicated in BBB breakdown and vasogenic edema) [29], and processes related to BBB disruption that may contribute to downstream tissue injury despite angiographic success [30]. In line with this biology, our peri-clot DE-CTA radiomic features derived from VNC and low-keV reconstructions may capture peri-clot low-attenuation heterogeneity and permeability-related microvascular changes compatible with edema or inflammatory activation (e.g., VNC_peri_glszm_SALGLE), whereas high-energy attenuation patterns may reflect chronic wall-adherent components potentially related to calcification or fibrosis (e.g., 190keV_peri_firstorder_Skewness).

Clot-centered DE-CTA radiomics captures clot phenotypes relevant to EVT, including attenuation (density), texture heterogeneity, and clot burden/shape. Prior work suggests fibrin/platelet-dominant clots tend to be mechanically stiffer and are associated with more difficult thrombectomy, whereas RBC-rich thrombi, which often exhibit a hyperdense artery sign on non-contrast CT, are more frequently linked to higher reperfusion success and better outcomes [31]. In addition, clot perviousness on CTA, an imaging surrogate of contrast penetration and microstructural properties, has been linked to both clot composition and treatment/outcome metrics [32, 33]. Consistent with these reports, our intra-clot signature contains attenuation-related surrogates (Mix/VNC intensity statistics), heterogeneity descriptors (GLCM/GLSZM/GLRLM/NGTDM features), and shape metrics (Flatness, LeastAxisLength), supporting that the model leverages clot density, heterogeneity, and size information.

The study had several limitations. First, its retrospective design may limit the applicability of the results, highlighting the need for prospective validation in a larger cohort. Second, the mechanistic interpretation of the peri-clot signature is limited by the absence of peripheral inflammatory biomarkers. We also lacked direct biological or vessel-wall validation to corroborate these imaging-based interpretations. Third, although radiomics offers strong interpretability through handcrafted, feature-level quantification, it may not capture more complex clot and peri-clot representations that could be learned by deep neural networks. Fourth, clinical adoption of DE-CTA may be constrained by availability, cost, and workflow integration; workflow time metrics were not systematically available in this retrospective cohort, and we therefore did not quantify its impact on time-to-puncture. Finally, we analyzed baseline pre-interventional DE-CTA only, and longitudinal post-EVT imaging could help characterize temporal changes in the peri-clot microenvironment and their association with recovery or deterioration.

## Conclusions

Radiomics of the tissue surrounding the clot on pre-interventional dual-energy CTA robustly predicts reperfusion and functional outcome after endovascular therapy. Models based on this peri-clot region outperform those using the clot alone or the clot together with its surroundings, confirming its unique value for risk stratification and treatment personalization.

## Supplementary information

ELECTRONIC SUPPLEMENTARY MATERIAL

## Data Availability

The data that support the findings of this study are not openly available due to reasons of sensitivity and are available from the corresponding author upon reasonable request. Anonymized data can be obtained for academic purposes by submitting a scientifically justified request to the corresponding author via institutional email.

## Abbreviations

AIS: Acute ischemic stroke
AUC: Area under the receiver operating characteristic curve
CI: Confidence interval
CT: Computed tomography
CTA: Computed tomography angiography
DCA: Decision curve analysis
DECT: Dual-energy computed tomography
DE-CTA: Dual-energy CT angiography
DSA: Digital subtraction angiography
EVT: Endovascular therapy
LASSO: Least Absolute Shrinkage and Selection Operator
LR: Logistic regression
Mix: Mixed-energy (DECT reconstruction)
mRS: Modified Rankin Scale
mTICI: Modified thrombolysis in cerebral infarction
NPV: Negative predictive value
PPV: Positive predictive value
ROC: Receiver operating characteristic
ROIs: Regions of interest
VM: Virtual monoenergetic
VNC: Virtual non-contrast

## Code availability

Code scripts can be obtained for academic purposes by submitting a scientifically justified request to the corresponding author via institutional email.
