# Supplementary material for: Dual-energy CT clot and peri-clot radiomics for predicting complete reperfusion and clinical outcome following endovascular therapy in acute ischemic stroke
Source: Insights Imaging. 2026 Mar 30;17:84. doi: 10.1186/s13244-026-02258-9 (PMC13036058; doi:10.1186/s13244-026-02258-9)
Supplement: Supplementary file 1 — ELECTRONIC SUPPLEMENTARY MATERIAL [file 13244_2026_2258_MOESM1_ESM.pdf]

Dual-Energy CT Clot and Peri-Clot Radiomics for Predicting Complete Reperfusion and Clinical Outcome Following Endovascular Therapy in Acute Ischemic Stroke

ELECTRONIC SUPPLEMENTARY MATERIAL

Figures

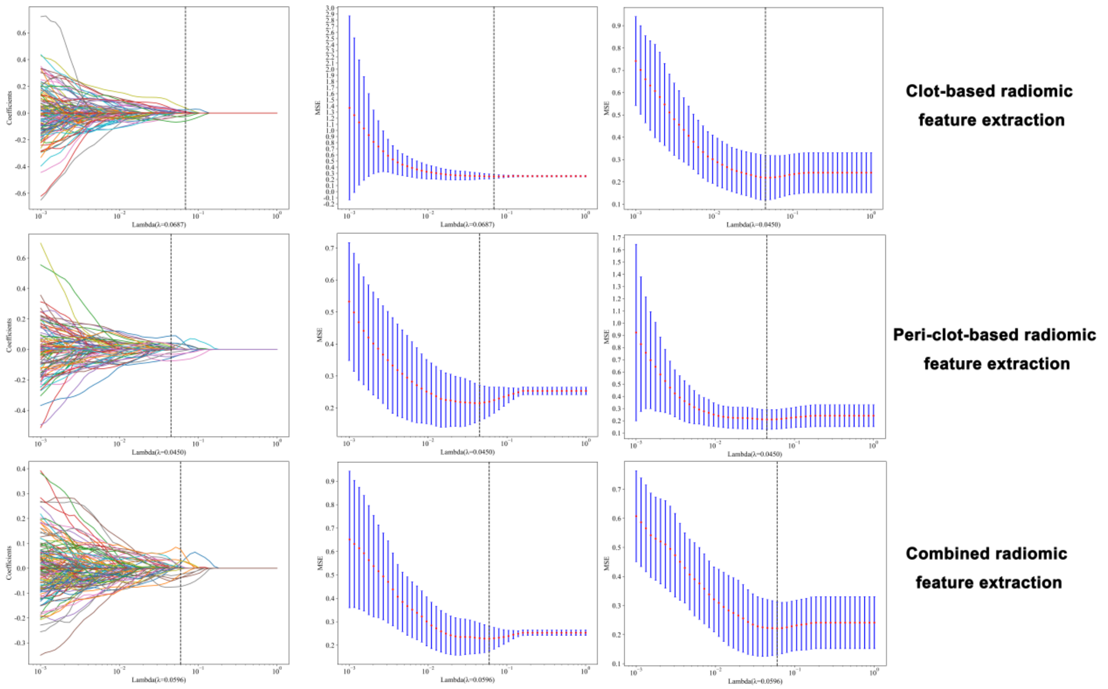

Fig. S1. Radiomics feature selection using the least absolute shrinkage and selection operator (LASSO). For each of the clot-based, peri-clot-based, and combined radiomics frameworks, the left column shows the LASSO coefficient profiles of all candidate radiomics features, the middle column presents the tuning parameter (lambda) selection using 10-fold cross-validation for the complete reperfusion model, and the right column presents the corresponding lambda selection curves for the good clinical outcome model. The optimal lambda values chosen by the minimum cross-validated error are indicated by vertical dashed lines, yielding the final subsets of non-zero coefficients used to construct the radiomics signatures.

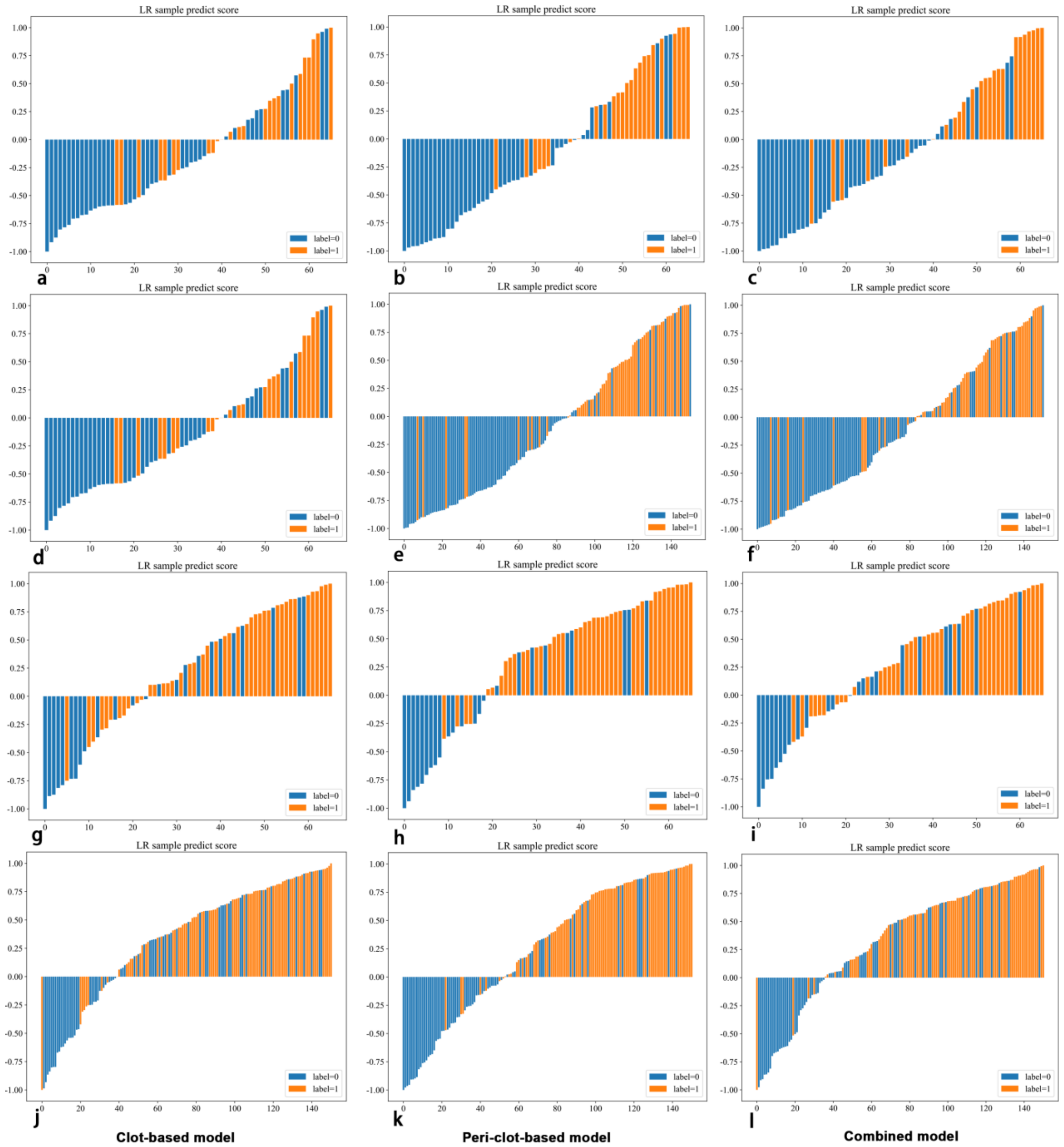

Fig. S2. Distribution of radiomics scores (rad-scores) for the clot-based, peri-clot-based, and combined models in individual patients. Each bar represents one patient; blue and orange bars denote patients with reference label 0 and 1, respectively. The left, middle, and right columns correspond to the clot-based, peri-clot-based, and combined models, respectively.

Panels (a–c) show model performance for predicting complete reperfusion (mTICI 3 vs ≤2b) in the internal cohort (train and test cohorts combined), and panels (d–f) show the corresponding results in the validation cohort. Panels (g–i) display model performance for predicting clinical outcome (90-day mRS 0–2 vs 3–6) in the internal cohort (train and test cohorts combined), and panels (j–l) display the corresponding results in the validation cohort.

For complete reperfusion, label = 0 indicates mTICI ≤2b and label = 1 indicates mTICI 3. For functional outcome, label = 0 indicates mRS 3–6 and label = 1 indicates mRS 0–2.

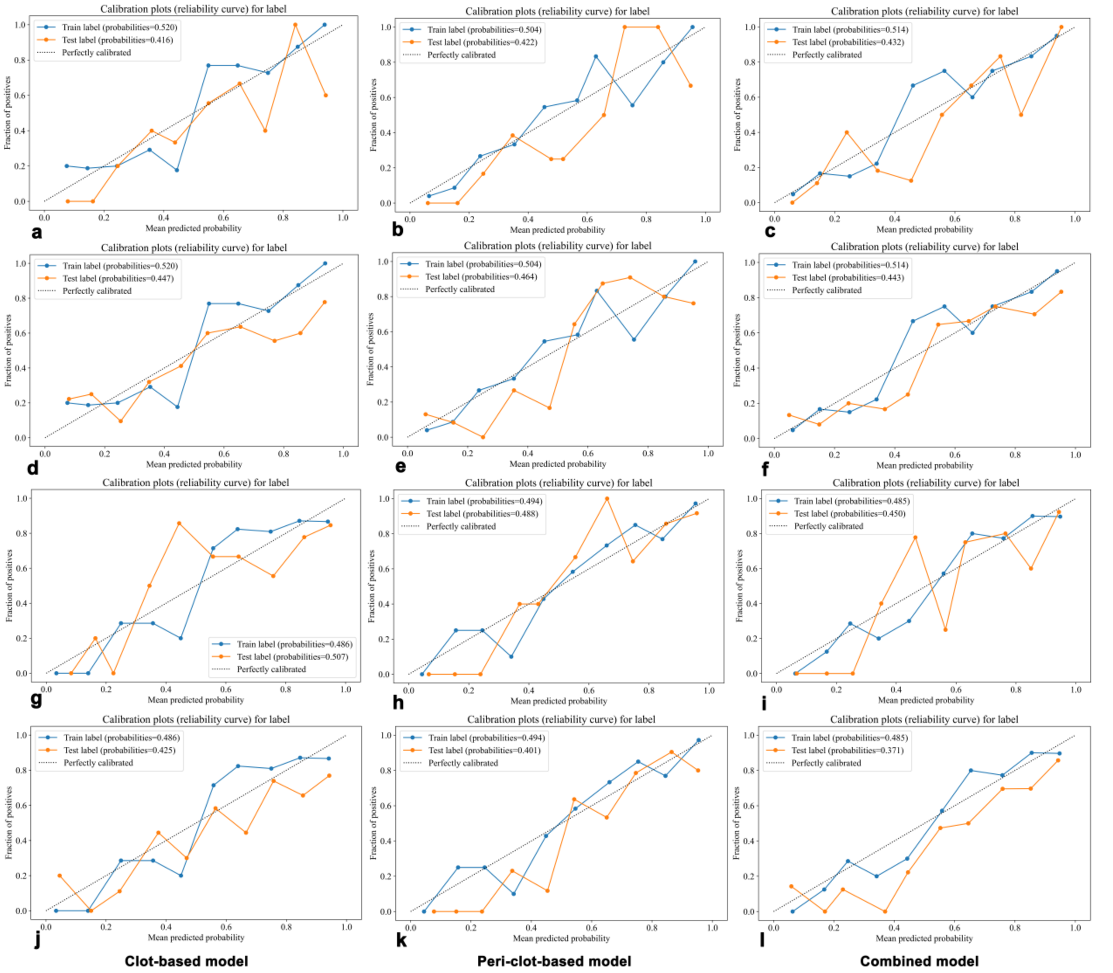

Fig. S3. Calibration curves across different models. The left, middle, and right columns correspond to the clot-based, peri-clot-based, and combined models, respectively. Panels (a–c) and (g–i) show calibration in the internal cohort (train and test cohorts) for complete reperfusion (mTICI 3 vs ≤2b) and good clinical outcome (90-day mRS 0–2 vs 3–6), respectively. Panels (d–f) and (j–l) show calibration in the internal and external validation cohorts. In the internal cohort, blue and orange lines represent the train and test cohorts, respectively. In the external validation cohort, blue and orange lines represent the internal and validation cohorts, respectively. The dashed diagonal line indicates perfect calibration.

Material and Methods

CT Scanning and EVT Procedures

Dual-energy capability was available on the baseline CT platform. Dual-energy acquisition was applied for CTA (DE-CTA), whereas baseline NCCT (and CTP, when performed) followed routine single-energy protocols, with standard mixed/blended CTA reconstructions available immediately for routine clinical interpretation. DE-CAT images were obtained using a dual-source CT scanner (SOMATOM Force; Siemens Healthineers) with settings: 128 × 0.6 mm, 0.25 s rotation, 0.7 pitch, and reference tube current-time product set to 90 mAs for the 90-kVp tube and 69 mAs for the Sn150-kVp tube. The reformatted section thickness was 1.0 mm with an increment of 0.7 mm. For CTA, patients received iodinated contrast media (Ultravist 370; Bayer Schering Pharma) via the peripheral antecubital vein at the rate of 1.5 mL/kg and 5 mL/s, followed by a 30 mL saline flush. DE-CTA-derived material decomposition images were generated as background post-processing and were not required for, or awaited during, acute clinical decision-making. The raw high- and low-keV DECT data were processed on the post-processing workstation (syngo. via VB20A, Dual Energy; Siemens Healthineers), reconstructed, and exported as Mix and Virtual Monoenergetic (VM, 40 keV and 190 keV, with a 20-keV interval), Virtual Non-Contrast (VNC), and Iodine Concentration (IC) images. IC images derived from DE-CTA based on a vendor-provided algorithm were designed for iodine applications.

All EVT procedures were conducted in accordance with institutional protocols for acute ischemic stroke management. The interventions were performed under either general anesthesia or conscious sedation, based on individual patient assessment. Thrombectomy strategies included stent-retrievers, contact aspiration, or combined approaches, with device selection at the operator's discretion. All neurointerventional devices were employed in strict compliance with their approved indications as regulated by the National Medical Products Administration (NMPA) of China.

Results
Radscore formulations

The prediction of complete reperfusion:
The clot-based model's radscore formulation:
label = 0.461038961038961 -0.013367 * 40keV_intra_original_firstorder_Skewness -0.025719 * 40keV_intra_original_glcm_InverseVariance -0.000316 * 40keV_intra_original_glszm_SmallAreaEmphasis +0.011608 * 40keV_intra_original_ngtdm_Busyness +0.038483 * Mix_intra_original_firstorder_Median +0.025108 * Mix_intra_original_glcm_SumAverage +0.007948 * Mix_intra_original_glrlm_RunLengthNonUniformity +0.000250 * IC_intra_original_glcm_ClusterShade +0.006389 * IC_intra_original_glszm_ZoneVariance -0.059599 * VNC_intra_original_glszm_LowGrayLevelZoneEmphasis +0.010709 * VNC_intra_original_ngtdm_Strength

The peri-clot-based model's radscore formulation:
label = 0.461038961038961 -0.056175 * 40keV_peri_original_firstorder_10Percentile -0.075185 * 40keV_peri_original_firstorder_Skewness -0.047545 * 40keV_peri_original_glcm_InverseVariance -0.020337 * 40keV_peri_original_ngtdm_Strength +0.008956 * 190keV_peri_original_firstorder_Skewness +0.013202 * 190keV_peri_original_glszm_GrayLevelNonUniformity +0.009632 * 190keV_peri_original_ngtdm_Strength +0.042575 * IC_peri_original_firstorder_Skewness +0.002018 * IC_peri_original_glszm_ZoneVariance +0.021308 * VNC_peri_original_firstorder_10Percentile +0.089263 * VNC_peri_original_firstorder_Median +0.037161 * VNC_peri_original_glcm_Correlation -0.002322 * VNC_peri_original_gldm_SmallDependenceLowGrayLevelEmphasis +0.003304 * VNC_peri_original_glszm_GrayLevelNonUniformity -0.031444 * VNC_peri_original_glszm_SmallAreaLowGrayLevelEmphasis +0.032022 * VNC_peri_original_shape_Flatness +0.006141 * VNC_peri_original_shape_LeastAxisLength

The combined model's radscore formulation:
label = 0.461038961038961 +0.004645 * IC_intra_original_glszm_ZoneVariance -0.037751 * VNC_intra_original_glszm_LowGrayLevelZoneEmphasis -0.025548 * 40keV_peri_original_firstorder_10Percentile -0.073310 * 40keV_peri_original_firstorder_Skewness -0.037994 *

40keV_peri_original_glcm_InverseVariance -0.003317 *
40keV_peri_original_ngtdm_Strength +0.031491 *
IC_peri_original_firstorder_Skewness +0.018833 *
VNC_peri_original_firstorder_Mean +0.076194 *
VNC_peri_original_firstorder_Median +0.010600 *
VNC_peri_original_glcm_Correlation -0.023887 *
VNC_peri_original_glszm_SmallAreaLowGrayLevelEmphasis +0.000484 *
VNC_peri_original_shape_Flatness +0.029353 *
VNC_peri_original_shape_LeastAxisLength

The prediction of good clinical outcome:

The clot-based model's radscore formulation:
label = 0.6558441558441559 -0.004909 *
40keV_intra_original_firstorder_Skewness -0.039288 *
40keV_intra_original_glcm_InverseVariance -0.041919 *
40keV_intra_original_gldm_SmallDependenceLowGrayLevelEmphasis -
0.041214 * 190keV_intra_original_ngtdm_Contrast +0.047338 *
Mix_intra_original_glcm_SumAverage -0.014857 *
Mix_intra_original_glrlm_ShortRunLowGrayLevelEmphasis +0.016181 *
IC_intra_original_glcm_MCC -0.006249 *
VNC_intra_original_firstorder_Minimum -0.013191 *
VNC_intra_original_glcm_Idm -0.010491 *
VNC_intra_original_glrlm_ShortRunLowGrayLevelEmphasis +0.004773 *
VNC_intra_original_shape_Flatness +0.080148 *
VNC_intra_original_shape_LeastAxisLength

The peri-clot-based model's radscore formulation:
label = 0.6558441558441559 -0.003002 *
40keV_peri_original_firstorder_10Percentile -0.005678 *
40keV_peri_original_firstorder_Minimum -0.021208 *
40keV_peri_original_firstorder_Skewness -0.057076 *
40keV_peri_original_glcm_InverseVariance +0.045534 *
190keV_peri_original_glszm_GrayLevelNonUniformity +0.005652 *
190keV_peri_original_ngtdm_Contrast +0.041664 *
IC_peri_original_glcm_Correlation +0.000220 *
VNC_peri_original_glszm_SizeZoneNonUniformity +0.108506 *
VNC_peri_original_shape_LeastAxisLength -0.001282 *
VNC_peri_original_shape_MinorAxisLength

The combined model's radscore formulation:
label = 0.6558441558441559 -0.019947 *
40keV_intra_original_glcm_InverseVariance -0.020554 *

40keV_intra_original_gldm_SmallDependenceLowGrayLevelEmphasis - 0.012361 * 190keV_intra_original_ngtdm_Contrast +0.032057 * Mix_intra_original_glcm_SumAverage -0.014531 * VNC_intra_original_glrlm_ShortRunLowGrayLevelEmphasis +0.006912 * VNC_intra_original_shape_LeastAxisLength -0.009359 * 40keV_peri_original_firstorder_Skewness -0.014806 * 40keV_peri_original_glcm_InverseVariance +0.022269 * 190keV_peri_original_glszm_GrayLevelNonUniformity +0.025828 * IC_peri_original_glcm_Correlation +0.078319 * VNC_peri_original_shape_LeastAxisLength

Table S1. Performance of clot, peri-clot, and combined models predicting complete reperfusion in non-tandem occlusions subgroup.

Cohorts	Models	SEN	SPE	ACC	PPV	NPV	Precision	Recall	F1	Threshold	AUC(95%CI)	P-value
Train cohort	Clot-based model	0.683	0.903	0.803	0.854	0.774	0.854	0.683	0.759	0.505	0.802 (0.721-0.880)	0.029[#]
	Peri-clot-based model	0.883	0.736	0.803	0.736	0.883	0.736	0.883	0.803	0.368	0.880 (0.825-0.931)	0.181[*]
	Combined model	0.867	0.750	0.803	0.743	0.871	0.743	0.867	0.800	0.385	0.862 (0.803-0.918)	0.071[^]
Test cohort	Clot-based model	0.750	0.725	0.734	0.621	0.829	0.621	0.750	0.679	0.455	0.769 (0.650-0.875)	0.088[#]
	Peri-clot-based model	0.958	0.650	0.766	0.622	0.963	0.622	0.958	0.754	0.342	0.855 (0.761-0.934)	0.638[*]
	Combined model	0.750	0.875	0.828	0.783	0.854	0.783	0.750	0.766	0.565	0.840 (0.725-0.930)	0.170[^]
Validation cohort	Clot-based model	0.692	0.691	0.692	0.590	0.778	0.590	0.692	0.637	0.439	0.718 (0.630-0.801)	<0.001[#]
	Peri-clot-based model	0.808	0.877	0.850	0.808	0.877	0.808	0.808	0.808	0.543	0.847 (0.768-0.914)	0.080[*]
	Combined model	0.808	0.802	0.805	0.724	0.867	0.724	0.808	0.764	0.489	0.822 (0.741-0.893)	0.005[^]
Overall	Clot-based model	0.713	0.746	0.733	0.664	0.787	0.664	0.713	0.688	0.439	0.760 (0.708-0.812)	<0.001[#]
	Peri-clot-based model	0.757	0.845	0.809	0.774	0.832	0.774	0.757	0.766	0.541	0.860 (0.816-0.899)	0.066[*]
	Combined model	0.801	0.767	0.781	0.708	0.846	0.708	0.801	0.752	0.466	0.843 (0.798-0.883)	<0.001[^]

SPE, Specificity; SEN, Sensitivity; ACC, Accuracy; PPV, Positive Predictive Value; NPV, Negative Predictive Value; AUC, Area Under the Receiver Operating Characteristic Curve;

CI, Confidence Interval. AUC 95% CI was estimated by stratified bootstrap resampling (2,000 resamples, percentile method).

[#], the clot-based model's AUC value compared with peri-clot-based model's by Delong test;

[*], the peri-clot-based model's AUC value compared with combined model's by Delong test;

[^], the combined model's AUC value compared with clot-based model's by Delong test.

Table S2. Performance of clot, peri-clot, and combined models predicting complete reperfusion in tandem occlusions subgroup.

Cohorts	Models	SEN	SPE	ACC	PPV	NPV	Precision	Recall	F1	Threshold	AUC(95%CI)	P-value
Train cohort	Clot-based model	0.909	0.818	0.864	0.833	0.900	0.833	0.909	0.870	0.580	0.876 (0.686-1.000)	0.425[#]
	Peri-clot-based model	0.909	0.909	0.909	0.909	0.909	0.909	0.909	0.909	0.447	0.926 (0.793-1.000)	0.252[*]
	Combined model	0.909	0.909	0.909	0.909	0.909	0.909	0.909	0.909	0.497	0.893 (0.727-1.000)	0.761^
Test cohort	Clot-based model	1.000	1.000	1.000	1.000	1.000	1.000	1.000	1.000	0.248	1.000 (1.000-1.000)	NA
	Peri-clot-based model	1.000	1.000	1.000	1.000	1.000	1.000	1.000	1.000	0.652	1.000 (1.000-1.000)	NA
	Combined model	1.000	1.000	1.000	1.000	1.000	1.000	1.000	1.000	0.505	1.000 (1.000-1.000)	NA
Validation cohort	Clot-based model	0.889	0.556	0.722	0.667	0.833	0.667	0.889	0.762	0.367	0.716 (0.444-0.926)	0.293[#]
	Peri-clot-based model	0.889	0.889	0.889	0.889	0.889	0.889	0.889	0.889	0.508	0.852 (0.617-1.000)	0.579[*]
	Combined model	0.667	1.000	0.833	1.000	0.750	1.000	0.667	0.800	0.593	0.815 (0.556-1.000)	0.436[^]
Overall	Clot-based model	0.667	0.810	0.738	0.778	0.708	0.778	0.667	0.718	0.580	0.771 (0.610-0.900)	0.085[#]
	Peri-clot-based model	0.905	0.857	0.881	0.864	0.900	0.864	0.905	0.884	0.447	0.884 (0.757-0.977)	0.251[*]
	Combined model	0.857	0.810	0.833	0.818	0.850	0.818	0.857	0.837	0.497	0.855 (0.717-0.959)	0.169[^]

SPE, Specificity; SEN, Sensitivity; ACC, Accuracy; PPV, Positive Predictive Value; NPV, Negative Predictive Value; AUC, Area Under the Receiver Operating Characteristic Curve; CI, Confidence Interval. AUC 95% CI was estimated by stratified bootstrap resampling (2,000 resamples, percentile method).

[#], the clot-based model's AUC value compared with peri-clot-based model's by Delong test;

[*], the peri-clot-based model's AUC value compared with combined model's by Delong test;

[^], the combined model's AUC value compared with clot-based model's by Delong test.

Table S3. Representative predictive models for thrombectomy-related procedural success and clinical outcomes: comparison of inputs, endpoints, and discrimination.

Study	Input	Endpoint	Reported AUC
Nishi et al., *Stroke* (2019) [1]	Pre-treatment clinical variables; ML (e.g., RF/LR/SVM)	clinical outcome	AUC (10-fold CV): RLR, 0.86±0.05; SVM, 0.86±0.06; RF, 0.85±0.07
Hofmeister et al., *Stroke* (2020) [2]	NCCT clot radiomics	First-attempt recanalization with thromboaspiration	Validation AUC 0.88
Xiong et al., *Quantitative Imaging in Medicine and Surgery* (2023) [3]	NCCT thrombus radiomics (ANN / multiple classifiers) ± clinical variables	Successful reperfusion (mTICI 2b/3)	Radiomics (ANN): Internal 0.873; External 0.805; Combined: Internal 0.860; External 0.849
Hilbert et al., *Comput Biol Med* (2019) [4]	CTA + deep learning	Functional outcome + recanalization	Functional outcome 0.71; recanalization 0.65 (reported averages)
Sommer et al. *Front Artif Intell*(2024) [5]	CTA + deep learning	Clinical outcome (3-month mRS)	Independent cohort: CTA, 0.70; CTA + Treatment, 0.86
Diprose et al. *J Neurointerv Surg*(2025) [6]	Baseline NCCT+CTA + deep learning	3-month functional outcome prediction	CT：Train, 0.83; Test, 0.69; Independent cohort, 0.69<br>CT + CTA：Train, 0.96 ; Test, 0.72; Independent cohort, 0.71
Zhang et al., *AJNR* (2024) [7]	Pre-treatment CT or MR + deep learning	First-pass effect	Prospective test: CT 0.81; MR imaging 0.80

This study	DE-CTA radiomics (peri-clot; VNC, 40 keV, 190 keV, etc.)	Complete reperfusion (mTICI 3)	Train 0.89; Test 0.86; External val 0.85
		Good clinical outcome (90-day mRS 0—2)	Train 0.83; Test 0.82; External val 0.85

This table summarizes selected machine-learning and deep-learning studies relevant to endovascular thrombectomy, alongside the present DE-CTA radiomics models. For each study, we report the primary imaging input(s), the predicted endpoint, and the area under the receiver operating characteristic curve (AUC) as reported by the original authors.

References：

1. Nishi H, Oishi N, Ishii A, et al (2019) Predicting Clinical Outcomes of Large Vessel Occlusion Before Mechanical Thrombectomy Using Machine Learning. Stroke 50:2379–2388. https://doi.org/10.1161/STROKEAHA.119.025411

2. Hofmeister J, Bernava G, Rosi A, et al (2020) Clot-Based Radiomics Predict a Mechanical Thrombectomy Strategy for Successful Recanalization in Acute Ischemic Stroke. Stroke 51:2488–2494. https://doi.org/10.1161/STROKEAHA.120.030334

3. Xiong X, Wang J, Ke J, et al (2023) Radiomics-based intracranial thrombus features on preoperative noncontrast CT predicts successful recanalization of mechanical thrombectomy in acute ischemic stroke. Quant Imaging Med Surg 13:682–694. https://doi.org/10.21037/qims-22-599

4. Hilbert A, Ramos LA, Van Os HJA, et al (2019) Data-efficient deep learning of radiological image data for outcome prediction after endovascular treatment of patients with acute ischemic stroke. Computers in Biology and Medicine 115:103516. https://doi.org/10.1016/j.compbiomed.2019.103516

5. Sommer J, Dierksen F, Zeevi T, et al (2024) Deep learning for prediction of post-thrombectomy outcomes based on admission CT angiography in large vessel occlusion stroke. Front Artif Intell 7:1369702. https://doi.org/10.3389/frai.2024.1369702

6. Diprose JP, Diprose WK, Chien T-Y, et al (2025) Deep learning on pre-procedural computed tomography and clinical data predicts outcome following stroke thrombectomy. J NeuroIntervent Surg 17:266–271. https://doi.org/10.1136/jnis-2023-021154

7. Zhang H, Polson JS, Wang Z, et al (2024) A Deep Learning Approach to Predict Recanalization First-Pass Effect following Mechanical Thrombectomy in Patients with Acute Ischemic Stroke. AJNR Am J Neuroradiol 45:1044–1052. https://doi.org/10.3174/ajnr.A8272
